# Enhanced Efficacy of Thiosemicarbazone Derivative-Encapsulated Fibrin Liposomes against Candidiasis in Murine Model

**DOI:** 10.3390/pharmaceutics13030333

**Published:** 2021-03-04

**Authors:** Azmat Ali Khan, Amer M. Alanazi, Nawaf Alsaif, Nasser Algrain, Tanveer Ahmad Wani, Mashooq Ahmad Bhat

**Affiliations:** Pharmaceutical Biotechnology Laboratory, Department of Pharmaceutical Chemistry, College of Pharmacy, King Saud University, Riyadh 11451, P.O. Box 2457, Saudi Arabia; saif_dna@yahoo.co.in (A.M.A.); nalsaif@ksu.edu.sa (N.A.); mailjabeen@yahoo.co.uk (N.A.); tawani@ksu.edu.sa (T.A.W.); mabhat@ksu.edu.sa (M.A.B.)

**Keywords:** fibrin liposome, *Candida albicans*, antifungal therapy, dual delivery, thiosemicarbazones

## Abstract

*Candida albicans* is the most studied species for the candidiasis infection and is becoming resistant towards existing antifungal drugs. Considering this, in the current study, we developed and characterized a fibrin liposome-based formulation encapsulating a novel thiosemicarbazone derivative, 2C, and evaluated its antifungal efficacy against murine candidiasis. The 2C-containing formulation was prepared by encapsulating 2C within the liposomes (2C-L) that were further encapsulated in the fibrin beads (2C-FL). The in-house synthesized 2C-FLs were spherical with a zeta potential of −34.12 ± 0.3 mV, an entrapment efficiency of 72.6 ± 4.7%, and a loading efficiency of 9.21 ± 2.3%, and they showed a slow and sustained release of 2C. Compared to free 2C, the formulation was non-toxic and exhibited serum stability, increased tissue specificity, and penetration. The 2C-FL formulation had a minimum inhibitory concentration (MIC) value of 4.92 ± 0.76 µg/mL and was able to induce apoptosis and necrosis in *C. albicans* in vitro. The administration of 2C-FL in *C. albicans*-infected mice prolonged their survival and antifungal effects when compared with the free 2C. The 2C-FL antifungal therapy significantly reduced the fungal burden and displayed an improved survival rate. In conclusion, the 2C thiosemicarbazone derivative possesses a potent antifungal activity that became more advantageous upon its encapsulation in the fibrin liposome delivery system.

## 1. Introduction

A dramatic increase in systemic fungal infections worldwide represents a serious health problem. An estimated 1.5 million people are affected by a fungal infection each year [1]. The increased incidences of these infections are manifested in immuno-compromised patients related to AIDS, cancer chemotherapy, organ transplant, or individuals with autoimmune diseases [2]. Candidiasis is the most prevalent fungal disease that includes a wide range of infections from superficial to systemic with high mortality rates [3,4]. It is caused in humans by the most common opportunistic fungi belonging to the genus *Candida*. *Candida* spp. is a multifaceted pathogen that causes mucosal, cutaneous, visceral, deep tissue, and disseminated infections [5]. During the past two decades, 85% of all fungal infections have been reported to be caused by *Candida albicans* [6,7]. *C*. *albicans*, along with some other *Candida* species, was highlighted as a serious threat by the Centers for Disease Control and Prevention’s (CDC) 2019 report [8]. For the treatment of infections caused by *Candida* spp., a range of antifungal drugs such as azoles, polyene, and echinocandins are available. Generally, due to their toxicity, low efficacy, serious adverse drug reactions, and acquired resistance, these drugs pose a hindrance in fulfilling medical needs [9,10,11,12]. The situation of the subsequent failure of existing therapeutics has necessitated the development of new antifungal agents that can combat the fungal infection with superior therapeutic efficacy.

Thiosemicarbazone derivatives have established their importance as a promising class of pharmacologically active candidates in the field of medicinal chemistry [13]. They possess abundant chemical adaptability and promising biological activities, such as antiparasitic [14], antiproliferative [15], anticonvulsant [16], antiviral [17], antimicrobial [18,19], and, particularly, antifungal [20]. In recent years, thiosemicarbazones have mostly been exploited for their antimicrobial and anticancer activities in the development of new drugs. Various studies have reported the antifungal activity of a series of thiosemicarbazone derivatives against *Candida* species [21,22,23]. Additionally, compounds bearing ligand recognition ability have been screened against *Candida* species. The antifungal activity of 4-arylthiosemicarbazides has been proposed to be possibly associated with the enzyme N-myristoyltransferase (NMT). From a class of thiosemicarbazide derivatives, 4-aryl/cyclohexyl thiosemicarbazides exhibit significant anti-Candidal activity [24,25,26,27]. Thiosemicarbazide derivatives bearing an isoquinoline ring have been reported to act against several *Candida* species [27]. We previously reported the synthesis, cytotoxicity, and antifungal activity of a series of N-(4-aryl/cyclohexyl)-2-(pyridine-4-yl carbonyl) hydrazinecarbothioamide derivatives in vitro [28]. From the series of substituted thiosemicarbazide derivatives, compound 2C was found to be the most effective one against *Candida albicans* ATCC 66027, Candida spp. 12,810 (blood) and Candida spp. The substitution of p-chloro at the phenyl ring of thiosemicarbazide resulted in enhanced anti-*Candida* activity. In particular, this compound showed similar to better activity than the reference drug (itraconazole) against several *Candida* strains. Compound 2C, bearing a p-chlorophenyl ring, was also the most potent derivative of the series against all strains of *Candida* spp. This could have been the result of increased lipophilicity associated with the p-chlorophenyl group. In the current study, we further evaluated the therapeutic efficacy of compound 2C against candidiasis in a murine model.

For the efficient and targeted delivery of drugs, the development of drug delivery vehicles has increased in recent years. New formulations with improved specificity and efficacy are being designed to enhance the therapeutic index of antifungal drugs. Studies have shown that the use of biodegradable polymers like poly-lactic-glycolic acid (PLGA), chitosan, and liposomes promotes slow-release and helps in reducing the toxicity and drug dosage that consequently helps to decrease side effects without compromising the fungicidal activity [29,30,31,32,33,34]. To date, various antifungal drugs have been administered in delivery vehicles. Amphotericin B encapsulated into multilamellar vesicles has been studied to reduce its toxicity and side-effects [35]. Likewise, nystatin encapsulated in nanoliposome has shown increased antifungal activity towards *C. albicans* than its free form in vitro [32]. The liposomal formulation of voriconazole has shown improved pharmacokinetics and a significant increase in anti-fungal activity towards *C. albicans* in mice [33]. Prolonged circulation time and improved overall targeting against *C. albicans* have been reported of itraconazole enveloped in polymeric nanoparticles [36]. It is worth mentioning that studies done in our laboratory using the novel fibrin microsphere system have shown great potential in the treatment of fungal infections. For instance, cytosolic proteins of *Cryptococcus neoformans* entrapped in a PLGA–fibrin bead dual delivery system showed an enhanced vaccine potential against experimental cryptococcosis in mice [29]. Treatment with amphotericin B-encapsulated fibrin microspheres [37] and the co-administration of amphotericin B and fluconazole entrapped in fibrin microspheres have shown better antifungal activity against *Cryptococcus* spp. [30]. This delivery system successfully released both large and small water-insoluble drugs in a sustained manner and decreased the fungal load within the organs in a mouse model. 

In the present study, we developed a dual delivery system containing liposomes and fibrin beads. We continued our research on the antifungal evaluation of compound 2C from series of N-(4-aryl/cyclohexyl)-2-(pyridine-4-yl carbonyl) hydrazinecarbothioamides. For the antifungal study, we used 2C in the fibrin liposomes delivery system and evaluated its fusogenic potential in efficiently contesting *C. albicans* infection in a mouse model.

## 2. Materials and Methods

### 2.1. Chemicals

Cholesterol, dimethyl sulfoxide (DMSO) and itraconazole were purchased from Sigma Aldrich, St. Louis, MO, USA. Human serum (Cat # 31876) was obtained from ThermoFisher Scientific, USA. Enzymes (creatinine, alkaline transferase (ALT), and total bilirubin) assay kits were procured from Span Diagnostics, India. A Vybrant Apoptosis Assay Kit #2 was bought from Molecular Probe (Eugene, Oregon). All chemicals used in the study were of the highest purity grade.

### 2.2. Animals

Female Swiss albino mice were used in the candidiasis study. Pathogen-free inbred Swiss albino mice (4–6 weeks in age with 20 ± 2 g of weight) were supplied by King Saud University, College of Pharmacy, Riyadh. The mice were housed in standard atmospheric conditions of 22 ± 1 °C temperature, 50–60% humidity, and a 12 h light–dark cycle. They were given water and diet ad libitum. All experiments were in accordance with the Guide for Experimental Animal Care Review Board (Ethics Reference No. KSU-SE-20-50), College of Pharmacy, King Saud University, Riyadh, Saudi Arabia.

### 2.3. Microbial Strain and Inoculum Preparation for Infection

A *C. albicans* (ATCC 18804) strain was procured from American Type Culture Collection, USA. The strain was stored at −80 °C and sub-cultured on Sabouraud dextrose agar plates or broth at 37 °C for 24 h. The fungal cells were washed thrice with sterile normal saline by centrifuging at 2000 rpm at 4 °C. Cell viability was determined by quantifying the cells with a hemocytometer. During the in vitro and in vivo studies, the cells were diluted in saline to the appropriate concentration.

### 2.4. Physico-Chemical Properties of Compound 2C

Compound 2C was from a series of N-(4-aryl/cyclohexyl)-2-(pyridine-4-yl carbonyl) hydrazinecarbothioamides that were previously synthesized in our lab [28]. IR (KBr) cm^−1^: 3414 (NH str.), 1663 (C=O str.), 1395 (C=S str.); ^1^H NMR (DMSO-*d_6_*): 7.3-8.7 (m, 8H, Ar-H), 9.9 (s, 1H, NH, D_2_O exchange), 10.8 (s, 1H, NH, D_2_O exchange, 11.1 (s, 1H, CONH, D_2_O exchange). ^13^C NMR (DMSO-*d_6_*): 206.0, 150.1, 128.5, 127.9, 121.6; HR MS: 259.2227 [M-46]^+^ [28].

### 2.5. Preparation of Samples

The tests were performed according to the standards proposed in the Clinical Laboratory Standard Institute (CLSI M27-A3) [38]. Compound 2C and itraconazole were dissolved in DMSO, where the final concentration of DMSO was less than 1.0%. The solutions were then filtered through 22 µm diameter filters.

### 2.6. Preparation of 2C-Encapsulated Liposomes

A 2C-encapsulated liposome (2C-L) was synthesized according to the previously published protocol of Khan et al. [39] with some standardization. An egg phophatidylcholine (PC) (49 µM) was mixed with 21 µM of cholesterol by sonication to form lipids. The lipids were combined with 2C (lipid: drug, 10:1 mol/mol) and dissolved in a round-bottomed flask in chloroform/methanol (1:1 *v/v*). The thin lipid film was formed on the wall of the flask by evaporating the solvents under a reduced pressure. The lipid film was now hydrated with phosphate-buffered saline (PBS) followed by sonication for 2 h in a bath-type sonicator at 4 °C. The traces of undispersed lipid were removed by high-speed centrifugation. The formed 2C-L formulation was washed three times with PBS to remove the non-entrapped solute. 

### 2.7. Preparation of 2C-Encapsulated Fibrin Liposomes

Fibrin beads were prepared by following the published protocol of Khan et al. [29]. Initially, 2C-L nanoparticles were mixed with 250 µL of plasma obtained from healthy mice. To this mixture, 40 mM calcium chloride was added, and then the aliquots from the reaction mixture were put as droplets over the parafilm-covered glass slide. To facilitate clot formation, the droplets were incubated at 37 °C for 40 min. The obtained formulation comprised 2C-encapsulated fibrin liposomes (2C-FL).

### 2.8. Characterization of 2C-FL

#### 2.8.1. Determination of Size and Surface Morphology

Particle size and surface morphology of 2C-L were evaluated by TEM. A drop of a lyophilized formulation in 20 mM PBS (pH 7.4) was mounted over gold-coated negative grid of a transmission electron microscope (Model HT 7700, Hitachi High Technologies, America Inc.) followed by the evaporation of the solvent. The analysis was performed at an accelerating voltage of 200 kV for their shape and morphology. The particle size of 2C-FL was determined using the optical microscope, and the mean surface diameter was determined on a millimeter scale.

#### 2.8.2. Determination of Zeta Potential

The zeta-potential of 2C-FL was examined using the Zetasizer Nano ZS (Malvern Instrument Limited, UK) as described earlier [30]. The samples were diluted in PBS (pH 7.4) to attain a suitable count rate. Zeta potential values were recorded with the DTS software for three independent experiments.

#### 2.8.3. Determination of Entrapment Efficiency and Loading Efficiency

The entrapment efficiency and loading efficiency were determined following the methods of Tang et al. [40]. Briefly, 10 mg of 2C-L and 2C-FL were separately mixed with 1 mL of methylene dichloride. The mixture was vigorously vortexed and then mixed into acetonitrile and deionized water (50v:50v). Next, methylene dichloride was evaporated using nitrogen stream, and the obtained clear solution was used for reverse-phase C_18_ column HPLC (Agilent Technologies, Waters, MA, USA). The column effluents were read by an ultraviolet detector at the λ_max_ of 306 nm to determine the 2C content in the liposomes and the fibrin liposome-based formulations. Results are expressed as the mean ± standard deviation (SD) of three independent experiments.

#### 2.8.4. In-Vitro Release Kinetics

The 2C release studies were performed according to the protocol of Khan et al. [30]. Briefly, an equal amount of 2C-FL was taken in multiple vials, and then one ml of PBS (20 mM, pH 7.4) was added to each vial. Release runs were incubated at 25 °C and continued for 5 days. Daily, 100 µL aliquot was taken from each vial and centrifuged at 10,000× *g* for 10 min. The supernatant of each sample was read at 306 nm, and the amount of released 2C was plotted against time in days. Results are expressed as the mean ± SD of three independent experiments.

### 2.9. Stability in Human Serum

The stability of 2C-L and 2C-FL was evaluated in the human serum. The formulation was mixed with human serum at the ratio of 1:9 *v/v*, and the reaction mixture was incubated at 37 °C. After 24 h, the incubation mixture was centrifuged at 5000× *g* for 15 min. The amount of 2C released from the formulation into the serum was calculated as a percentage of total compound 2C in the formulation added to the serum at the initial time. Results are expressed as the mean ± SD of three independent experiments.

### 2.10. Pharmacokinetics and Biodistribution Studies

For pharmacokinetic studies, 2C-L and 2C-FL were administered to healthy mice. A single dose of 5 mg/kg body weight (b.w.) of the formulations was injected via the tail vein. Free 2C was administered in a single dose comprising 1 mg/kg b.w. Blood aliquots were collected using a non-mortality model [41] at different periods, and serum was centrifuged out. The highest concentration of a drug (C_max_) was derived from plasma-concentration versus time profiles, and the other key pharmacokinetic parameters like the area under the plasma concentration versus time curve from 0 to 24 h (AUC_0–24_) and plasma clearance (CL) were calculated according to non-compartmental analysis [42]. Results are reported as the mean ± SD of three independent experiments.

The biodistribution study was done on mice after 12 h of receiving a single dose of 5 mg/kg b.w. intraperitoneally. The lung, liver, and kidney of the mice treated with 2C-L and 2C-FL were rapidly excised and washed with ice-cold saline. Tissues were blotted dry, weighed, and stored at −80 °C until further analysis. Compound 2C concentration in tissues was determined by HPLC (Agilent Technologies, Waters, MA, USA). Results are expressed as the mean ± SD of three independent experiments.

### 2.11. Toxicity Tests

The in vitro toxicity of free 2C and 2C-FL was determined using the previously published method of erythrocyte lysis test [39]. Briefly, blood samples from a healthy rabbit were collected in an anticoagulant solution. The samples were centrifuged at 4 °C, the pellet containing erythrocytes was washed with PBS (pH 7.4), and, finally, 50% hematocrit was prepared. Then, 0–10 mg/ml of free 2C, 2C-F, 2C-L, and 2C-FL were separately mixed with 0.2% red blood corpuscles (RBC) suspension and incubated at 37 °C for 1 h. The hemolytic effects were evaluated by visible spectroscopy at 576 nm, and percent hemolysis was calculated. The 100% hemolytic ability of Triton X-100 was taken as the positive control. Results are expressed as the mean ± SD of three independent experiments.

For in vivo toxicity, renal and hepatic toxicities were evaluated in healthy mice. According to the method of Khan et al., [39], a three-dose regimen of 400 µg formulation/dose/mouse was intraperitoneally given on the 1st, 3rd, and 5th days. Blood samples from different treatment groups were taken as pre-dose (a day before the administration of the formulation) and post-dose (the 6th day of the administration of the formulation). The levels of serum creatinine, ALT, and total bilirubin were evaluated by employing a commercial detection kit. Results are expressed as the mean ± SD of three independent experiments. Moreover, the extent of toxicity of 2C-FL was determined on liver and kidney histopathology.

### 2.12. Histopathological Examination of Tissue Sections

Mice from the 2C-L- and 2C-FL-treated groups were euthanized, and liver and kidney tissues were collected and prepared according to the published protocol with some modification [43]. Briefly, the fixed tissues were dehydrated and embedded in paraffin wax. The paraffin sections were kept at 37 °C for 14 h. Next, paraffin from the sections was removed by three consecutive washings in xylol. The sections were then dehydrated in series of alcohol grades (70–100%), and, finally, the sections were stained with hematoxylin and eosin and visualized under a light microscope (Olympus CLX 41) at 200X magnification.

### 2.13. In Vitro Testing of 2C-FL against C. albicans Infection

#### 2.13.1. Determination of Minimum Inhibitory Concentration (MIC)

Antifungal susceptibility was determined by broth microdilution method, where the lowest concentrations of antifungal agent showing a 50% minimum inhibitory effect on *C. albicans* growth compared to the growth of untreated control was determined [30]. Fungus (2 × 10^3^ cells/ml) was exposed to different concentrations of 2C-L and 2C-FL from 0.1–1 µg/mL diluted in Rosewell Park Memorial Institute (RPMI) 1640 medium (pH 7.0) buffered with 4-morpholine propanesulfonic acid (MOPS) in a 96-well microtiter plate. Concentrations of 10 µL of free 2C (1 µg/mL), 100 µL of 2C-L (10.6 µg/mL equivalent to 1 µg/mL 2C), and 100 µL of 2C-FL (10.2 µg/mL equivalent to 1 µg/mL 2C) were separately used for incubating with *C. albicans.* The RPMI 1640 medium only and DMSO only were taken as negative controls, whereas fungus in RPMI 1640 was taken as a positive control. The plates were incubated for 24 h at 37 °C. The turbidity of *C. albicans* was measured at absorbance of 570 nm, and the MIC of the compound and drug against the fungus was determined from two independent experiments performed in triplicates and expressed as mean ± SD.

#### 2.13.2. Annexin-V Assay for Detecting Apoptosis

The therapeutic effect of 2C-FL against fungal infection was done by detecting the presence of apoptosis markers in *C. albicans*, as mentioned by Tang et al. [40]. Briefly, 5 × 10^6^ colony-forming units (CFU)/mL of *C. albicans* were grown at 37 °C for 5 h in the RPMI 1640 medium containing 2C-L and 2C-FL, separately. The culture was further incubated for 48 h at 37 °C with shaking at 180 rpm. After harvesting and washing with PBS (pH 7.4), the fungus was digested with a lysis buffer. After digestion, apoptotic cell death was analyzed using an annexin V assay as per the published protocol [44]. The fungal cell suspension was incubated with annexin V-FITC (50 µg/mL) and propidium iodide (PI; 200 µg/mL) in dark for 20 min at room temperature. The apoptotic and necrotic cells were scored by flow cytometry (MACSQuant, Germany).

### 2.14. The Activity of 2C-FL against C. albicans Infection in a Mouse Model

#### 2.14.1. Determination of 2C Dose for Treatment

The dose of 2C for the antifungal treatment in vivo was determined according to the already published report [37]. Five groups (*n* = 5/group) comprising healthy mice were intraperitoneally injected with different doses of 2C. Mice of the sixth group (control group) were intraperitoneally injected with PBS. The mortality rate of each group was recorded after 24 h, and the number of deceased mice was scored for the calculation of median lethal dose (LD_50_).

#### 2.14.2. Treatment Schedule

The antifungal activity of 2C-FL was determined in a mouse model with *C. albicans* infection. For infection, healthy mice were infected with an inoculum of 100 µL saline containing 5 × 10^5^ CFU of *C. albicans* per mouse by an intravenous route in the lateral tail vein. One day post-infection, the mice were randomly distributed into six groups (*n* = 10/group) and treated with formulations (2 mg 2C/kg b.w.) for five days. Infected mice in group 1 received no treatment, group 2 mice were treated with sham FLs, group 3 mice were treated with free 2C, group 4 mice were treated with 2C-F, group 5 mice were treated with 2C-L, and group 6 mice were treated with 2C-FL. A simultaneous set of experiments was conducted for the survival studies.

#### 2.14.3. Fungal Burden Studies

The antifungal activity of 2C-FL against *C. albicans* infection was evaluated by assessing the clearance of fungal burden from treated mice. Briefly, on the 7th, 14th, and 21st days of post-infection, the fungal burden was determined in the liver, lung, and kidney of treated mice. Each organ was homogenized in 5 mL of PBS, and different dilutions of the homogenate were plated on Sabouraud dextrose agar plates. Plates were incubated at 37 °C for 48 h, and CFUs were counted and *C. albicans* load was calculated as a log of protection [30].

#### 2.14.4. Survival Studies

The antifungal ability of 2C-FL was also determined by evaluating the survival rate of the treated mice. The study was conducted for 60 days, during which mice were monitored for mortality twice each day and results were scored.

### 2.15. Statistical Analysis

A one-way ANOVA was applied for data analysis followed by Student’s *t*-test. *p* < 0.05 values were taken as statistically significant. Data analysis was done using SPSS v20.0 software.

## 3. Results

### 3.1. Physico-Chemical Properties of 2C

**Scheme 1:**
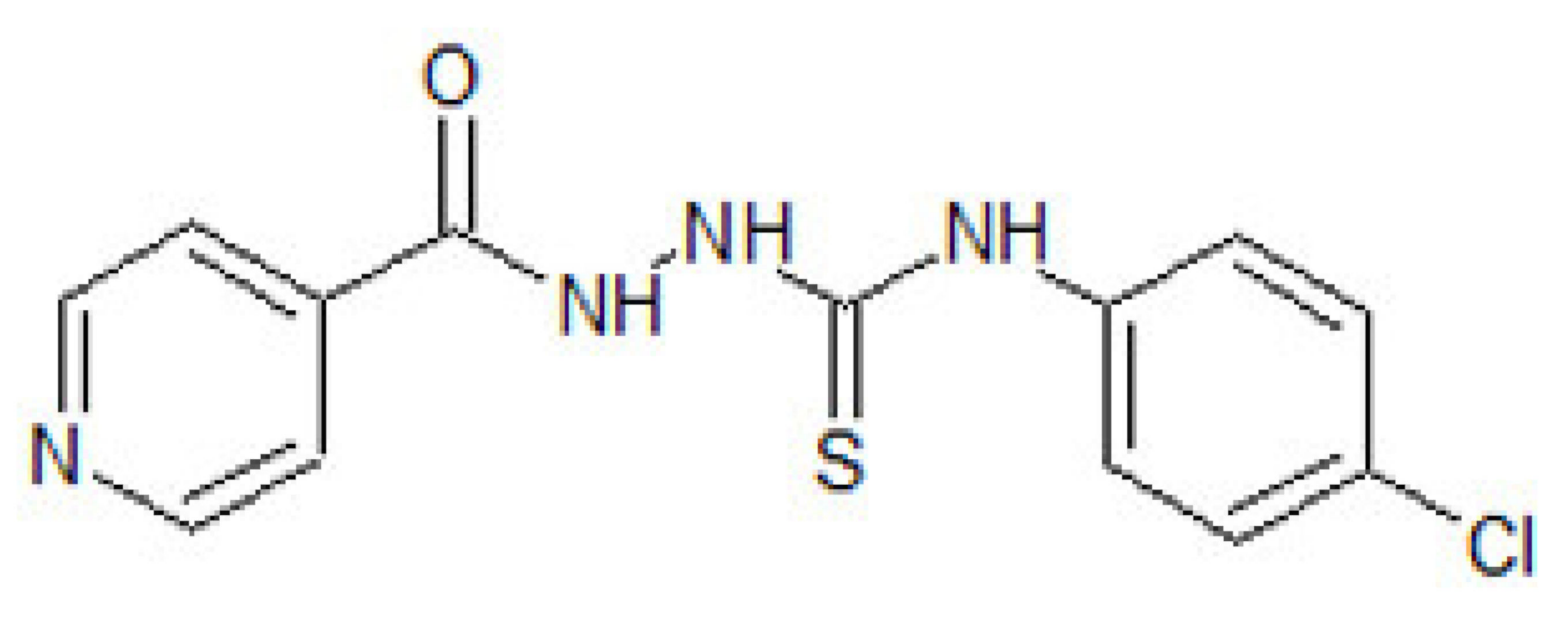
Structure of thiosemicarbazone derivative 2C.

The chemical structure of 2C is shown in Scheme 1. The physico-chemical properties of 2C have been analyzed before [28,45]. The purity of the compounds was checked by thin layer chromatography and elemental analysis. The compounds were characterized and confirmed by spectral data. The compound was also checked for its solubility and thermodynamics parameters [45].

### 3.2. Characterization of 2C-FL

**Figure 1:**
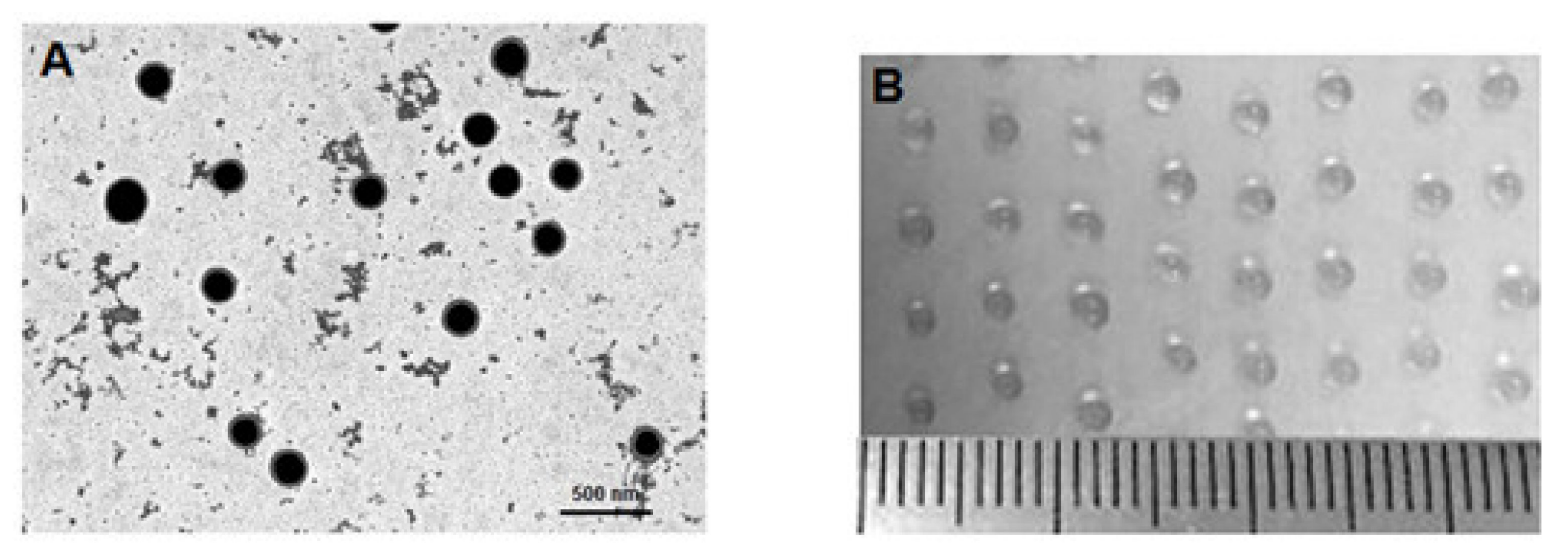
Physical properties of 2C-FL (2C-encapsulated fibrin liposomes) formulations. (**A**) Transmission electron microscopic image of 2C-L nanoparticles. (**B**) Representative optical image of 2C-FL formulations.

Morphology and size: The TEM image of 2C-liposomes (2C-L) exhibited a smooth and spherical shape with an average size of 200–300 nm (Figure 1A; Table 1). After the further encapsulation of 2C-L into the fibrin beads (2C-FL), their size changed. As observed from an optical image, 2C-FLs were spherical, with an average size of 1.0–1.3 mm (Figure 1B; Table 1).

Zeta potential: The dispersion stability of any nanoparticle is exhibited by its surface charge, which is determined by an important predictor, zeta potential. The average zeta potential of 2C-FL was −34.12 ± 3.3 mV, whereas the zeta potential of 2C-L was −23.35 ± 1.2 mV (Table 1).

Entrapment efficiency: As shown in Table 1, the % entrapment efficiency of 2C-FL was 72.6 ± 4.7% and that of 2C-L was 78.5 ± 5.2%. The % loading efficiencies of 2C-L and 2C-FL were 9.63 ± 1.2 and 9.21 ± 2.3, respectively. The high % entrapment efficiency of fibrin liposomes suggested them to be an ideal delivery vehicle for drugs.

### 3.3. In-Vitro Release of 2C-FL

**Figure 2:**
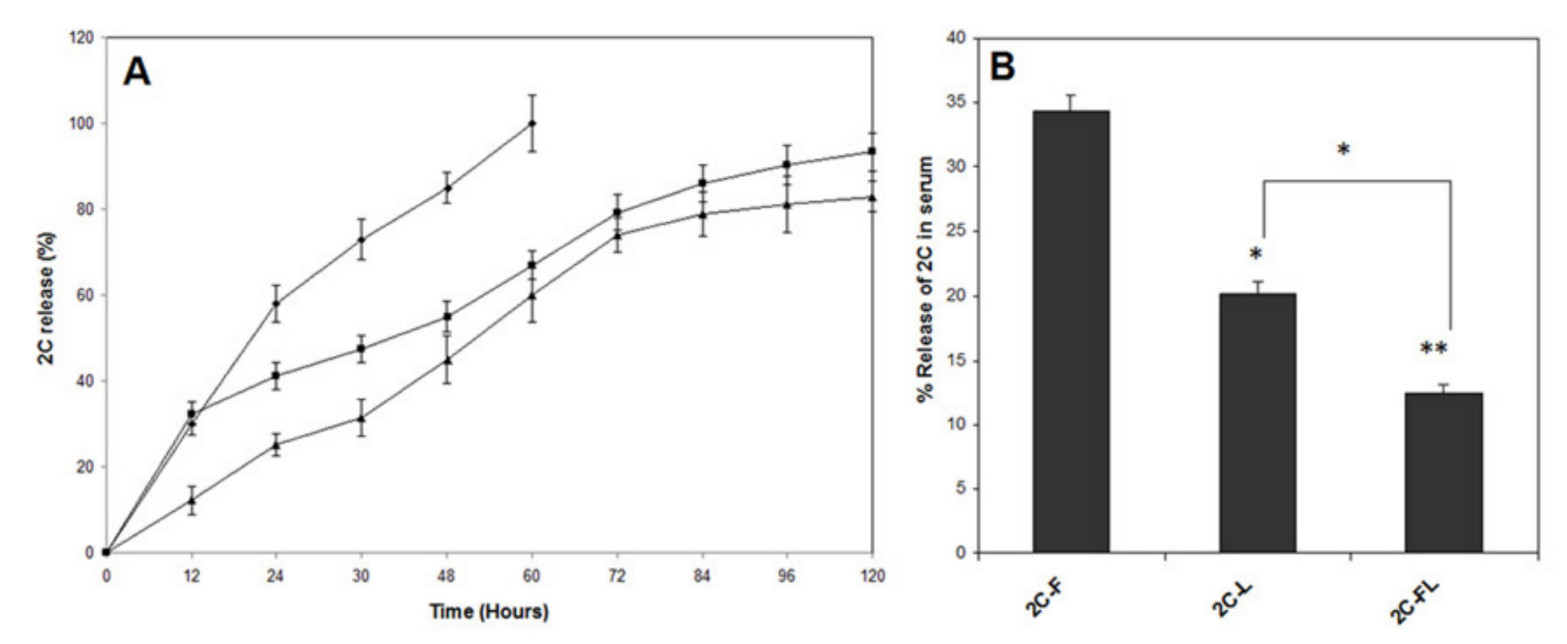
Cumulative in vitro release of 2C from different types of formulations. (**A**) Profile showing the kinetic release of 2C from the different formulations *viz.*, 2C-F, 2C-L and 2C-FL. (**B**) Profile showing the release of 2C from the different formulations *viz.*, 2C-F, 2C-L and 2C-FL into the serum after 24 h. Data are presented as the mean ± standard deviation (SD) of three independent experiments. * *p* < 0.01; ** *p* < 0.001.

The release kinetics of 2C from freshly prepared formulations was evaluated during the time period of 120 hours. As shown in Figure 2A, 82.9 ± 6.4% of 2C were released from the fibrin liposome formulation in a slow and sustained manner. In contrast to this, almost 32.4 ± 2.8% of 2C were released in the form of initial burst from liposome nanoparticles, followed by slow and constant release (93.2 ± 7.2%). Both of the formulations released the encapsulated drug in the time span of 120 h. Fibrin beads were unable to sustain the compound for a longer duration and showed a 100% release within 60 h.

### 3.4. Stability of 2C-FL in Human Serum

Figure 2B shows the percent stability of 2C-FL in human serum. The incubation of the formulation with human serum resulted in the slow release of the compound. Compared to 2C-L, 2C-FLs were more stable in human serum, showing a 12.3% leakage of 2C from the formulation into the serum. The 20% leakage of 2C in the serum was detected from the liposomes, whereas 34.4% was released from the fibrin beads.

### 3.5. Pharmacokinetics and Tissue Distribution of 2C-FL

A distinct difference was observed in the pharmacokinetic parameters of 2C from the fibrin liposome formulation. The concentration of the compound was higher in the systemic circulation of mice treated with 2C-FL compared to those treated with 2C-F or 2C-L (Table 2). The encapsulation of 2C into the fibrin liposome reduced the clearance of 2C by almost half and increased the AUC_0–24_ by 3-fold. After 20 min of the administration of 2C-FL and 2C-L, the C_max_ values of 2C were 6.81 ± 2.5 and 5.32 ± 1.8 µg/mL, respectively. The mean AUC_0–24_ of 2C-FL was 5.94 ± 3.7 µg/mL x h in comparison to the AUC_0–24_ values of 2C-L (4.35 ± 2.3 µg/mL x h). 2C-FL showed a decline in clearance (CL = 43.31 ± 8.4 mL/h) compared to 2C-L (CL = 58.34 ± 5.2 mL/h). In comparison to 2C-FL and 2C-L, the 2C-F formulation showed a low C_max_ value, suggesting a lower concentration in the systemic circulation. Moreover, the encapsulation of 2C in the fibrin beads showed an increased clearance compared to 2C-FL and 2C-L.

**Figure 3:**
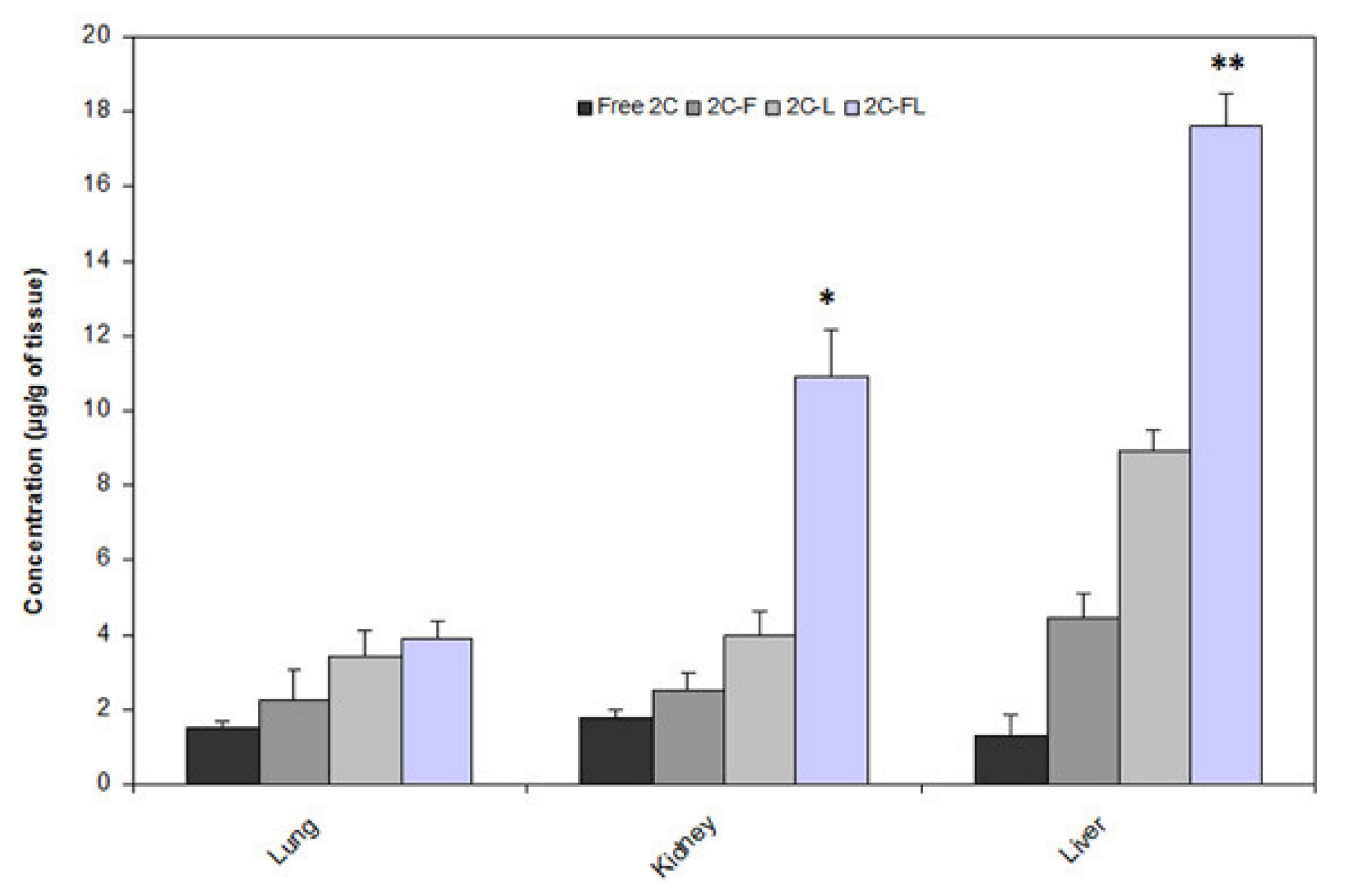
Biodistribution profile of 2C from fibrin beads, liposomes, and fibrin liposomes. Distribution of 2C after 12 h of intraperitoneal administration of free 2C, 2C-F, 2C-L, and 2C-FL. Data are presented as the mean ± SD of three independent experiments. Free 2C versus 2C-F, 2C-L, or 2C-FL; * *p* < 0.05; ** *p* < 0.01.

To ensure the pharmacokinetic values obtained from blood concentrations, the quantification of 2C-FL was investigated as biodistribution in lung, liver, and kidney tissues after 12 h of drug administration (Figure 3). 2C-FL was observed in significantly high concentrations in the liver (*p* < 0.01) when compared with free 2C. The concentration of 2C-FL in the kidney was also significantly high (*p* < 0.05) in comparison to free 2C, but the overall concentration was less than that found in the liver. In the lung, the 2C-FL was present in a smaller quantity than in the liver and kidney. Compared to free 2C, a significantly high concentration of 2C-L was observed in the liver, but almost comparable concentrations of 2C-L were present in the lung and kidney. The 2C-F formulation was present in a lesser quantity in the lung and kidney compared to 2C-L and 2C-FL.

### 3.6. 2C-FL Toxicity In Vitro and In Vivo

**Figure 4:**
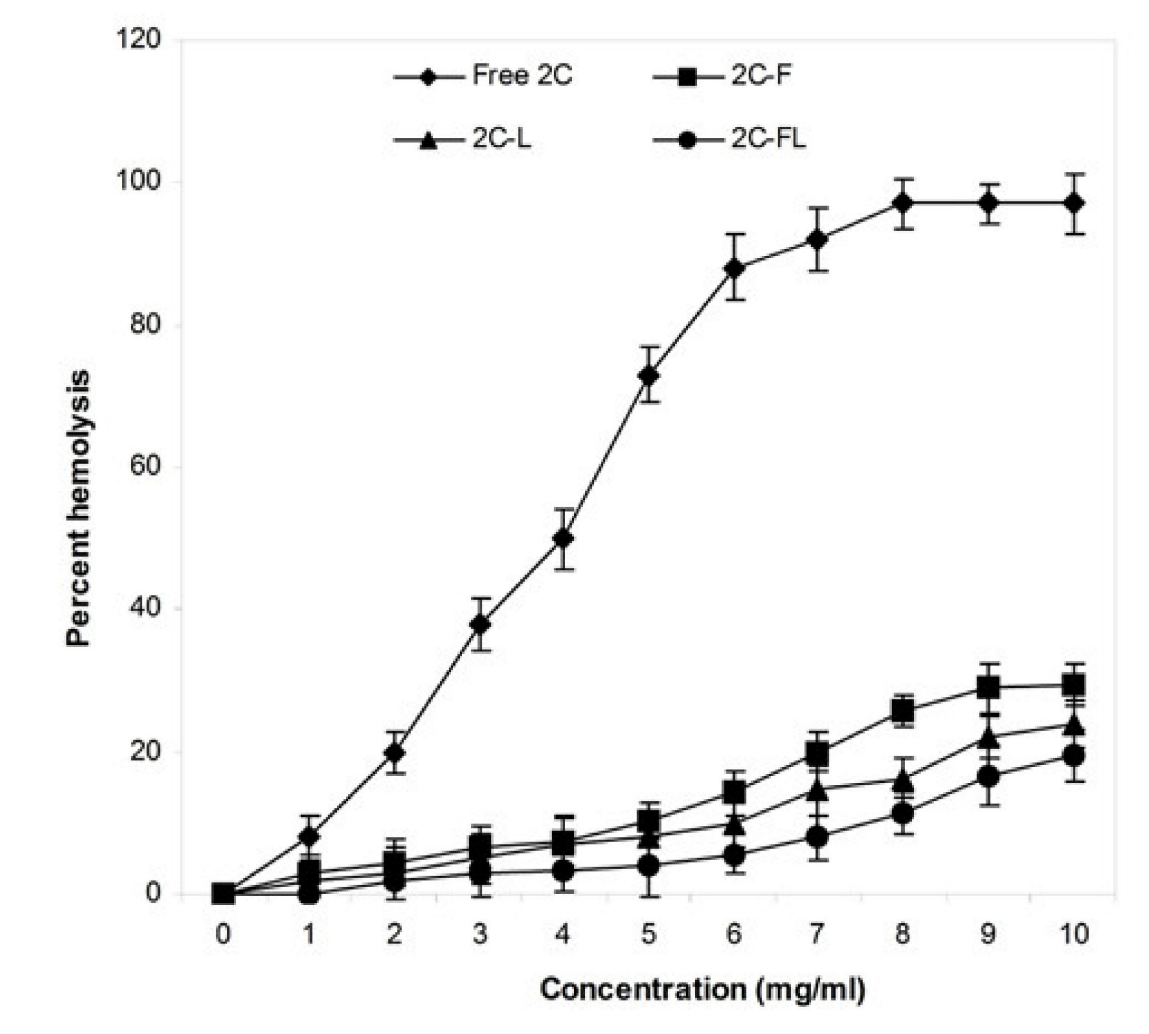
Percent hemolysis of erythrocytes caused by 2C-F, 2C-L, and 2C-FL formulations. Erythrocytes from the healthy rabbit were incubated with different concentrations of free 2C, 2C-F, 2C-L, and 2C-FL for 1 h at 37 °C. Data are presented as the mean ± SD of three independent experiments.

The hemolytic ability of free 2C, 2C-L, and 2C-FL was observed in a dose-dependent manner (Figure 4). Free 2C showed a 100% hemolytic ability to erythrocytes at a dose of 10 mg/L. However, the encapsulation of 2C within liposomes and fibrin liposomes showed a significant reduction in its hemolytic ability. Compared to free 2C, 2C-FL showed only a 19.6% hemolytic ability to erythrocytes at a dose of 10 mg/L (Figure 4). The other two formulations, i.e., 2C-F (29.4%) and 2C-L (24%), also showed less hemolytic activity to erythrocytes in comparison to free 2C.

In vivo toxicity parameters showed that there were no alterations in the hematological profile or the weight of the organs after six days. The changes in the biochemical parameters caused before day one and on the sixth day after the last dose administration are given in Table 3. The 2C-FL group showed no statistical differences in the creatinine levels compared to the healthy control values (*p* < 0.05). Both the ALT and bilirubin levels also did not present a statistical difference in the 2C-FL-treated group. Similarly, there was no obvious change in the levels of serum creatinine, ALT, and bilirubin values in the 2C-F and 2C-L groups. Regarding the hematological values of the free 2C group mice, although the levels of serum creatinine, ALT, and bilirubin showed an increase in comparison to the control group, the values were within the expected values of healthy mice.

**Figure 5:**
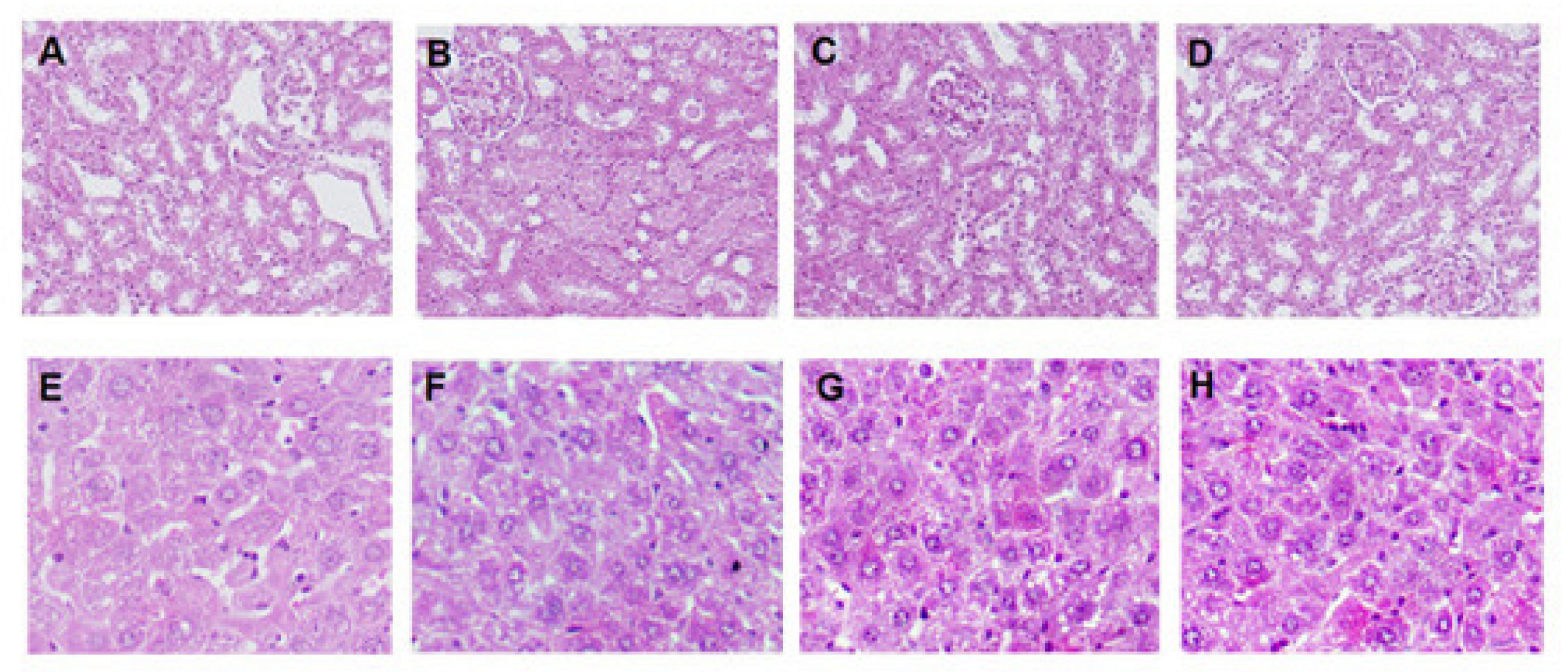
The extent of toxicity visualized on kidney and liver tissue sections of healthy mice after administering 2C-L and 2C-FL formulations. Representative histopathological images of kidney sections: (**A**) free 2C, (**B**) 2C-F, (**C**) 2C-L, and (**D**) 2C-FL; liver sections: (**E**) free 2C, (**F**) 2C-F, (**G**) 2C-L, and (**H**) 2C-FL at 200 × magnification.

As far as the toxic effect of 2C-FL on tissue histopathology is concerned, there was no substantial change in the architecture of the kidney (Figure 5A–D) and liver (Figure 5E–H) tissues. As can be seen in Figure 5D, the renal parenchyma was almost of normal architecture in 2C-FL-treated mice. Normal renal corpuscles showing glomerular mass and cellularity were quite distinct. Moreover, both proximal and distal convoluted tubules were identified by their intact linings. Likewise, 2C-L did not affect the general morphology of the tissue (Figure 5C). A mild toxic effect was observed after 2C-F treatment (Figure 5B), whereas a prominent toxicity was manifested on the free 2C-treated section (Figure 5A). Free 2C caused a pathological change in the form of distorted glomeruli and dilated tubules. In the liver, 2C-FL and 2C-L did not produce any significant changes, (Figure 5H,G, respectively) and the liver tissue section was very much akin to the normal architecture. Hepatic laminae and sinusoids were visible with no known hepatocyte necrosis, fibrosis, or lymphocytic infiltration. 2C-F treatment resulted in enlarged sinusoids (Figure 5F). The histopathological change caused by free 2C showed focal lytic necrosis and enlarged sinusoids (Figure 5E).

### 3.7. Activity of 2C-FL against C. albicans Infection In Vitro

As shown in Table 4, the MIC value of 2C was calculated to be 0.46 ± 0.05 µg/mL against *C. albicans* infection. Considering the formulation to compound ratio (10:1 mol/mol), 2C-FL with an MIC value of 4.92 ± 0.76 µg/mL contains 0.492 µg/mL of 2C. This concentration of 2C was approximately equivalent to the MIC value obtained for free 2C. This thereby indicates that 2C-FLs possess an antifungal activity similar to free 2C and that fibrin liposome did not affect the antifungal activity. Itraconazole was observed to exhibit inhibitory activity, with an MIC of 1.26 ± 0.71 µg/mL. The calculated MIC value of itraconazole was similar to the already reported value [46].

**Figure 6:**
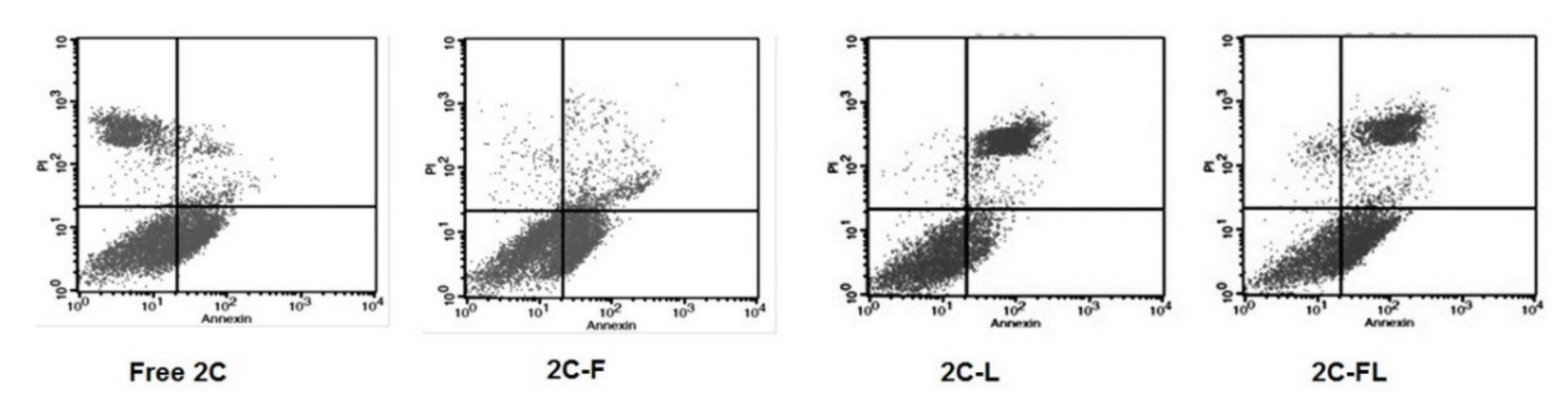
In vitro antifungal activity of 2C-encapsulated formulations against *C. albicans* using annexin V-FITC staining. Cell apoptosis was measured in fungal cells after exposing them to free 2C, 2C-F, 2C-L, and 2C-FL. The upper left quadrant of the flow cytometry density plot represents the necrotic cells (annexin^−^  propidium iodide (PI)^+^), the upper right quadrant represents late apoptotic cells (annexin^+^ PI^+^), the lower right quadrant represents early apoptotic cells (annexin^+^ PI^+^), and the lower left quadrant represents viable cells (annexin^+^ PI^−^).

The inductions of apoptosis and necrosis were used as the criteria to investigate the antifungal activity of 2C-FL on *C. albicans*. The presence of apoptotic and necrotic cells was detected by annexin V-FITC staining. As shown in Figure 6, 2C-FL showed early apoptosis (43.82%), late apoptosis (27.01%), and necrosis (2.4%). 2C-L-treated cells also showed the percentage of apoptotic and necrotic cells. In contrast, 2C-F and free 2C treatment showed the population of early apoptotic cells. There was a prominent increase in the necrotic cells after treatment with free 2C. Dot plot results from the 2C-FL treatment group demonstrated that the fungal cell population was positive for apoptosis, as well as necrosis.

### 3.8. Therapeutic Efficacy of 2C-FL against C. albicans Infection

#### 3.8.1. Dose of 2C for In Vivo Treatment

To use the novel compound 2C in in vivo administration, firstly, its dose was determined. After treating the healthy mice with different doses of 2C for 24 h, the mortality LD_50_ value calculated was 20 mg/kg b.w. One-tenth of the LD_50_ value, i.e., 2 mg/kg b.w., did not show any mortality even after three weeks of treatment. Hence, this dose was taken for the antifungal therapy in mice. The selected dose was similar to the already reported dose of thiosemicarbazones [47].

#### 3.8.2. Fungal Burden in Treated Mice

**Figure 7:**
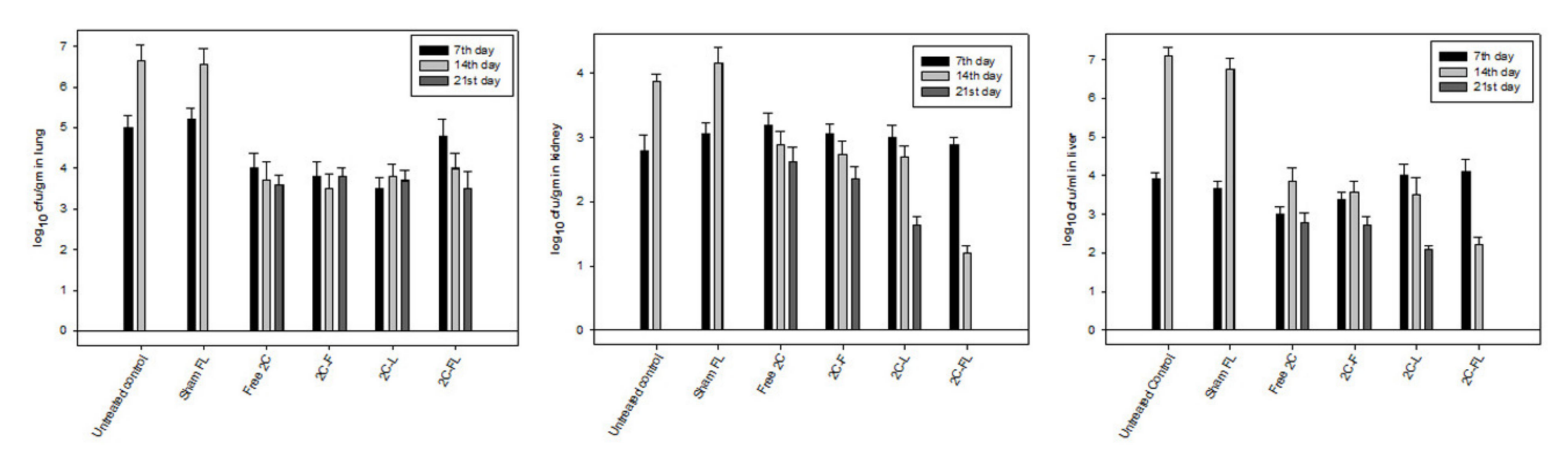
Antifungal efficacy of 2C-encapsulated formulations on the fungal burden in C. *albicans-*-infected mice. Antifungal activity was detected as a reduction in fungal load at the 7th, 14th, and 21st days post-infection. Data are presented as the mean ± SD CFU/organ of three independent experiments.

The antifungal efficacy assessed by fungal burden depicted a significant reduction of *C. albicans* load in the lung, liver, and kidney of infected mice after 2C-FL therapy. As shown in Figure 7, the 2C-FL and 2C-L formulations showed significant inhibitory activity against the fungal growth in tested organs compared to the untreated control. The maximum reduction of fungal burden was observed in the liver (4.1 ± 0.33–2.2 ± 0.18 log CFU/g; *p* < 0.01) followed by the kidney (2.9 ± 0.10–1.2 ± 0.10 log CFU/g; *p* < 0.05), and after, 21 days of treatment, there were no fungi present in the liver and kidney of 2C-FL-treated *C. albicans*-infected mice. Additionally, 2C-FL significantly reduced the fungal burden in the lung (4.8 ± 0.41–3.5 ± 0.43 log CFU/g; *p* < 0.05) when compared with the untreated control. Significant reductions in the fungal burden of 4.0 ± 0.28–2.1 ± 0.17 log CFU/g in the liver (*p* < 0.01), 3.5 ± 0.26–3.7 ± 0.24 log CFU/g in the lung, and 3.0 ± 0.18–1.6 ± 0.13 log CFU/g (*p* < 0.05) in the kidney were observed in 2C-L-treated *C. albicans*-infected mice, but the decrease of fungal burden was not comparable to the 2C-FL formulation. The reduction in fungal burden in 2C-F-treated *C. albicans*-infected mice was visible with high effect in the liver (3.4 ± 0.18–2.72 ± 0.22 log CFU/g) and kidney (3.04 ± 0.14–2.33 ± 0.19 log CFU/g), but the formulation was not as effective as the 2C-FL- and 2C-L-treated groups. Likewise, free 2C also reduced the fungal burden with a high effect in the liver (3.0 ± 0.19–2.8 ± 0.23 log CFU/g) and kidney (3.2 ± 0.18–2.6 ± 0.23 log CFU/g) compared to the untreated control group. Infected mice from the untreated control group showed an increase in infection till the 14th day. The same case was observed for the sham FL-treated *C. albicans*-infected mice, where the fungal burden increased and all mice showed mortality till the 15th day.

#### 3.8.3. Survival of Treated Mice

**Figure 8:**
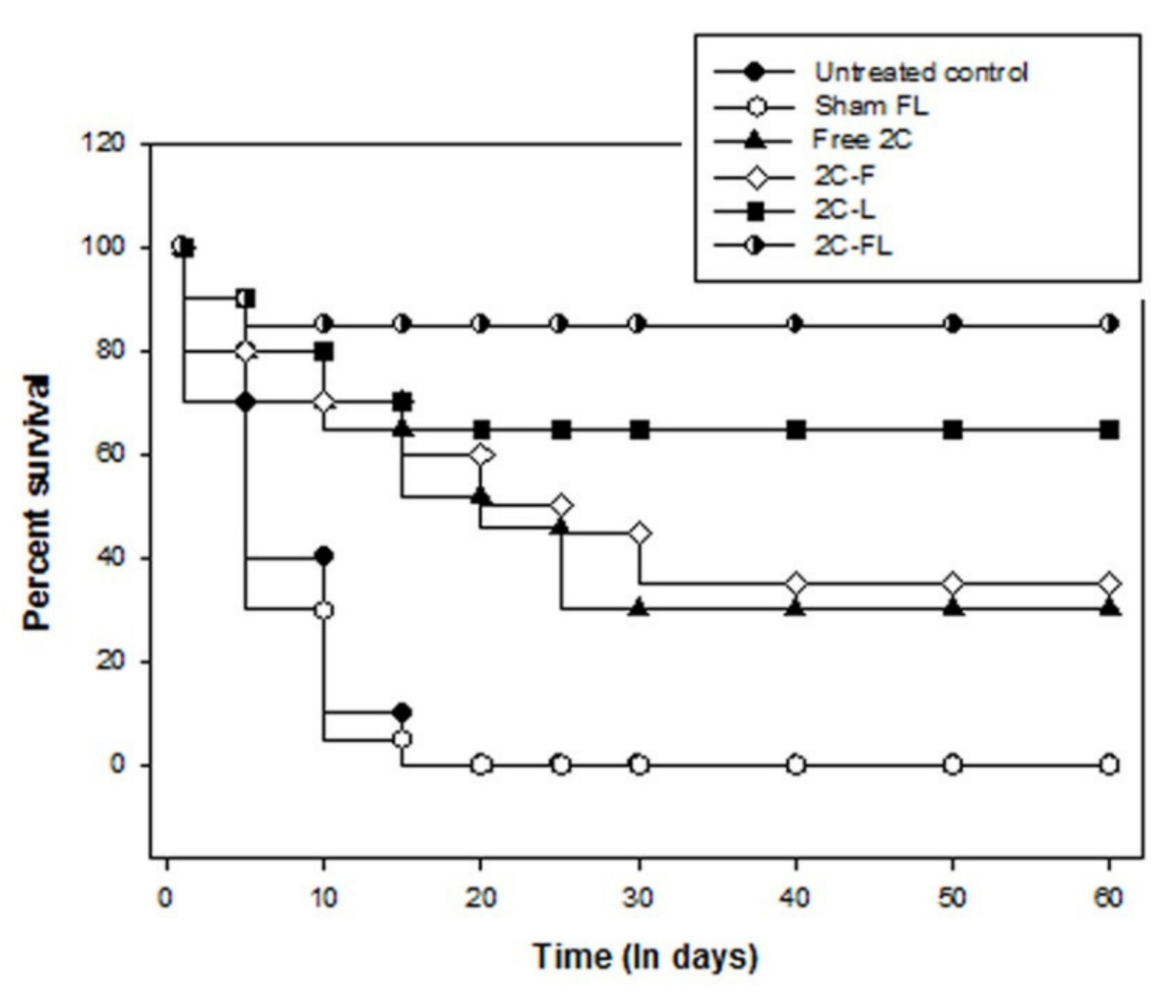
The survival rate of *C. albicans*-infected mice after the treatment with 2C-encapsulated formulations. Survival of mice was recorded for 60 days post-infection.

The survival data of *C. albicans* infected mice after therapy are shown in Figure 8. The group of *C*. *albicans*-infected mice receiving no treatment (untreated control) was not able to survive after 15 days of the survival study. As can be seen from the plot, the best regression of mortality was observed in mice treated with the 2C-FL formulation. Unlike untreated control, almost 85% of the treated mice survived till day 60 of post-infection. The treatment of infected mice with the 2C-L formulation showed a distinct efficacy, where almost 65% of animals survived till day 60. The treatment with 2C-F showed a 35% survival of treated mice till the 30th day, and free 2C showed a 30% survival of treated mice till the 30th day. Sham FL and the untreated mice survived till the 15th day.

## 4. Discussion

The therapeutic efficacy of antifungal drugs is achieved by utilizing different types of approaches. Unfortunately, the exposed cells become resistant and acclimatize to drug pressure due to the similar fungi-static effects of these drugs [48]. The conventional mode of drug administration also does not help in increasing the antifungal effect and leads to low bioavailability, poor drug release, and retention. Nowadays, advanced drug delivery systems are applied as practical strategies to overcome the issues of the current treatments. Drugs entrapped in delivery vehicles are protected from earlier degradation and consequently show a longer half-life and decreased side-effects [49]. The use of a drug delivery system helps in the controlled release of a drug to a specific site for an extended time period, subsequently increasing its efficacy [50]. In our earlier study, we presented the substituted thiosemicarbazide compound 2C as a newer, potent, and promising antifungal agent showing minimal toxicity to non-cancer cell lines and remarkable anti-fungal activity against *Candida* spp. [28]. Herein, we tried to encapsulate the compound 2C into fibrin liposome formulation and to evaluate its antifungal ability against experimental candidiasis in Swiss albino mice. *C. albicans* was chosen because it is the most prevalent opportunistic fungi causing infections in humans [3].

The promising role of liposome nanoparticles can be seen in the latest available therapeutic drug AmBisome^®^, which has shown improved efficacy and reduced toxicity against fungal infection in clinical settings [51] and which is one of the most commonly used liposomal formulations for the treatment of systemic *Candida* infections [52]. Liposomal formulations have shown enhanced skin permeation and have increased the stability of topically-used antifungal drugs [53]. The use of plasma beads as a delivery vehicle offers the possibility where both small/large molecules can be entrapped—there is no need for any additional proteins or enzymes and they help in the successful delivery of various therapeutic agents in the surrounding milieu [29]. Fibrinogen is an important constituent of plasma that is converted to fibrin during blood coagulation and forms a 3D network due to polymerization. Naturally, the clotting process is catalyzed by thrombin. Various studies have reported the adhesive properties of the fibrin in the entrapment of pharmacological agents and the controlled release of the agents like antibiotics, enzymes, and other substances in the surrounding milieu [54]. The preparation of fibrin beads from autologous whole plasma offers an exceptional benefit of minimizing the risk of immunological complications. Moreover, the clotting of plasma is achieved by activating the endogenous thrombin by adding CaCl_2_. Hence, by considering the characteristic potential of both liposome and plasma beads and being inspired by the advantages of already reported fibrin microspheres [30,37], we used the dual delivery system of fibrin liposome in this study. Moreover, since 2C is insoluble in water, the use of fibrin liposome as a delivery vehicle was also considered to overcome the technical issues faced during the development of an injection.

The pre-requisites of any biodegradable nanoparticles are their size, surface, and stability, which play key roles in the release of the drug and its cellular uptake, internalization, and in vivo pharmacokinetics [55]. Size, zeta-potential, and % entrapment efficiency represent the physicochemical characteristics of a nanoparticle. The dispersion ability of any nanoparticle is determined by its surface charge or zeta potential. It is an important predictor that influences its stability in a solution and its interaction with the cell membrane. The high % entrapment efficiency suggests a high affinity between the nanoparticle and the drug. In the present study, the as-synthesized 2C-FLs were homogenously spherical, with an average size of 1.0–1.3 mm (Figure 1B). The zeta-potential, % entrapment efficiency, and % loading efficiency were within a favorable range, thus indicating a beneficial encapsulation of 2C to fibrin liposomes that also helped in the release of 2C from fibrin liposomes in a slow and sustained manner. This pattern of slow and sustained release suggested 2C to be entrapped in the core and matrix of fibrin liposomes. Our release kinetic data were in accordance with already reported results. Overall, the increased release of 2C is an add-on to the therapeutic efficacy of fibrin liposome formulations. It can be suggested that in-house designed 2C-FL has the potential to rapidly internalize in cells and release 2C in a slow and sustained manner.

For the determination of in vivo antifungal activity of 2C-FL, knowledge of its stability, toxicity, pharmacokinetics, and biodistribution is vital. The stability of any nanoparticle has always been an important factor for its application. In the present study, the stability of 2C-FL was determined by its incubation with human serum. After 24 h of incubation, only 12.3% of 2C was released from fibrin liposome formulations into the serum, thus indicating increased stability in comparison to the release of 2C from the liposomal formulation and fibrin beads (Figure 2B). The increased stability of the 2C-FL formulation could be because of the presence of fibrin beads that protect the lipid bilayer from the action of serum proteins. The pharmacokinetics of any drug can be modulated after its encapsulation into a nanoparticle. For example, the modification of blood circulation time or tissue distribution could change the efficiency of a drug [56]. The pharmacokinetic results indicated the presence of 2C for an extended time period in the systemic circulation of mice treated with 2C-FL compared to those treated with 2C-L and 2C-F. The pharmacokinetic parameters depicted that fibrin liposomes reduced the clearance of 2C and increased the AUC_0–24_ (Table 2). The liver and kidney are the prime target organs where fungal species colonize [57], and the colonization of *C. albicans* changes the permeability of blood vessels and results in the accumulation of nanoparticles by passive targeting (Bazak et al., 2014; Sercombe et al., 2015). In our biodistribution studies (Figure 3), there was an increased accumulation of 2C in the liver and kidney of mice treated with 2C-FL compared to those treated with 2C-L. It can be suggested that the increased accumulation of 2C-FL at these infection sites might result in the superior antifungal activity of 2C.

We also investigated the toxicity level of 2C-FL in vitro and in vivo. For in vitro studies, erythrocytes were incubated with 2C-FL and 2C-L. It was observed that free 2C was able to induce erythrocyte lysis to some extent (Figure 4). However, the toxicity of 2C was abrogated after its encapsulation within fibrin liposomes, as well as in liposomes or fibrin beads. The results were in concordance with the in vivo toxicity results, where 2C-FL did not produce any significant change in the serum creatinine, ALT, and bilirubin levels in healthy mice (Table 3). The toxicity results were similar to our earlier studies with amphotericin B and fluconazole-fibrin microspheres [30]. The histological results also supported the anti-toxic effect of 2C-FL when it did not produce any renal and hepatic toxicity (Figure 5). Liver and kidney tissue sections were akin to the normal architecture, with no detectable changes. These results thus showed that the encapsulation of 2C in fibrin liposomes reduced the toxicity of 2C and protected the animal cells from toxicity while providing antifungal activity.

Preliminary antifungal susceptibility testing showed the potent antifungal activity of 2C-FL against *C. albicans*. Quantitatively, the presence of a similar dose of 2C in 2C-FL to that of free 2C (MIC value) signified that 2C possesses antifungal activity and there is no antifungal role of the fibrin liposomes (Table 4). The compound showed a better activity than the reference drug itraconazole for the *Candida* strain, and this best activity was observed due to the presence of the *p*-chlorophenyl group [28]. Recent studies have indicated the presence of apoptosis in yeast induced by different agents [58,59]. Dying *C. albicans* cells have been reported to exhibit chromatin condensation, nuclear fragmentation, and DNA damage—the key markers of apoptosis [60,61]. In the present study, we also tried to detect the presence of apoptosis in 2C-FL-treated *C. albicans*. The externalization of phosphatidylserine is widely considered to be a marker of apoptosis. Annexin V can bind to phosphatidylserine and is used as a basis to detect apoptotic cells. Herein, the 2C-FL treatment caused phosphatidylserine externalization and membrane disruption in fungal cells, as evidenced by annexin V staining. The formulation was able to induce apoptosis and necrosis that could be predicted to lead to *C. albicans* cell death (Figure 6).

The therapeutic efficacy of 2C-FL was found to be manifested in *C. albicans*-infected mice by comparing the antifungal effects in different organs and the survival of infected mice after therapy. The fungal burden was determined by detecting the reduction in the CFU/g of *C. albicans* in the lung, liver, and kidney. As shown in Figure 7, a significant reduction in CFU count was observed in all the three tested organs after 2C-FL therapy. The antifungal efficacy of 2C-FL was observed till 21 days, after which no effect was observed on the residual fungus. Compared to the control group, the 2C-FL-treated group showed the most pronounced regression of *C. albicans* infection in the kidney and liver, where no fungi were observed on the 21^st^ day. The amount of *C. albicans* was reduced in the 2C-F-, 2C-L-, and free 2C-treated groups; however, the decrease in fungal burden was not comparable to that of the 2C-FL-treated group. The survival rate of *C. albicans*-infected mice was significantly better (85%) in the 2C-FL-treated group when compared with the other treated groups. *C. albicans*-infected mice showed a 100% mortality after 15 days in the untreated control group. Interestingly, these results indicated the presence of the 2C-FL formulation for a longer time period that, in turn, can be attributed to its high stability. All the therapeutic augmentation data ascribed the antifungal efficacy of 2C-FL over other formulations and also demonstrated the superiority of the fibrin liposome-based delivery system.

## 5. Conclusions

The novel 2C-encapsulated fibrin liposomes proved to be an unconventional formulation that showed significant antifungal efficacy in the clearance of *C. albicans* infection in mice. The entrapment of 2C in fibrin liposomes helped to enhance the fungicidal ability of the compound. The developed 2C-FLs were of suitable size and zeta potential, and they showed a high level of 2C encapsulation. They were non-toxic, exhibited extended and controlled release, and were able to remain stable in serum over time. The delivery vehicle was able to provide protection, selective penetration, and accumulation in the affected tissues. The in vivo results clearly demonstrated that 2C-FL had a significantly higher antifungal activity against *C. albicans* compared to the free form of the compound. This is the first-ever report using a novel thiosemicarbazone derivative encapsulated in fibrin liposome formulations against *C. albicans*. Additionally, a specific mechanism of antifungal therapy should be investigated.

## Figures and Tables

**Table 1:** Characteristics of 2C-encapsulated formulations.

Formulations	Particle Size	Zeta Potential (mV)	Entrapment Efficiency (%)	Loading Efficiency (%)
2C-L	200–300 nm	−23.35 ± 1.2	78.5 ± 5.2	9.63 ± 1.2
2C-FL	1.0–1.3 mm	−34.12 ± 3.3	72.6 ± 4.7	9.21 ± 2.3

**Table 2:** Pharmacokinetic parameters of 2C in the blood of healthy mice after the intraperitoneal administration of 2C-F, 2C-L, and 2C-FL nanoparticles. Data are presented as the mean ± SD of three independent experiments. Free 2C versus 2C-L or 2C-FL; * *p* < 0.05. C_max_: highest concentration of a drug; AUC_0–24_: area under the plasma concentration–time curve from 0 to 24 h; CL: plasma clearance.

Formulations	C_max_ (µg/mL)	AUC_0–24_ (µg/mL x h)	CL (mL/h)
Free 2C 2C-F 2C-L 2C-FL	1.4 ± 0.25 2.8 ± 0.44 5.32 ± 1.8 * 6.81 ± 2.5 *	0.91 ± 0.17 1.79 ± 0.32 4.35 ± 2.3 * 5.94 ± 3.7 *	76.63 ± 7.7 66.73 ± 7.2 58.34 ± 5.2 43.31 ± 8.4

**Table 3:** Serum levels of creatinine, aminotransferase alanine (ALT), and bilirubin were evaluated in healthy mice after the administration of a single dose of 2C-F, 2C-L, and 2C-FL nanoparticles. Data are presented as the mean ± SD of three independent experiments.

		Healthy Control	Free 2C	2C-F	2C-L	2C-FL
Creatinine (mg%)	Day 0 Day 6	0.28 ± 0.07 0.29 ± 0.04	0.29 ± 0.06 0.34 ± 0.05	0.28 ± 0.03 0.31 ± 0.02	0.27 ± 0.03 0.30 ± 0.05	0.28 ± 0.02 0.26 ± 0.03
ALT (U/I)	Day 0 Day 6	40.5 ± 6.5 40.2 ± 4.2	40.9 ± 7.3 45.1 ± 8.5	41.1 ± 5.6 42.6 ± 6.2	39.9 ± 6.6 41.4 ± 5.3	40.3 ± 8.2 40.9 ± 5.9
Total Bilirubin	Day 0 Day 6	0.16 ± 0.06 0.18 ± 0.03	0.15 ± 0.04 0.21 ± 0.07	0.15 ± 0.07 0.20 ± 0.04	0.17 ± 0.02 0.19 ± 0.08	0.16 ± 0.01 0.17 ± 0.05

**Table 4:** In vitro antifungal activity of free 2C and 2C-encapsulated formulations against *C. albicans* as determined from minimum inhibitory concentration (MIC) values.

Formulations	MIC (µg/mL)
Free 2C 2C-F 2C-L 2C-FL	0.46 ± 0.05 2.63 ± 0.18 5.11 ± 0.32 4.92 ± 0.76

## Data Availability

Not applicable.
